# The *B*RAzil *MAG*nesium (*BRAMAG*) trial: a randomized clinical trial of oral magnesium supplementation in pregnancy for the prevention of preterm birth and perinatal and maternal morbidity

**DOI:** 10.1186/1471-2393-14-222

**Published:** 2014-07-08

**Authors:** Joao Guilherme B Alves, Carla Adriane Fonseca Leal de Araújo, Isabelle E A Pontes, Angélica C Guimarães, Joel G Ray

**Affiliations:** 1 Department of Paediatrics, Instituto Materno Infantil Prof. Fernando Figueira-IMIP, Recife, Brazil; 2 Dom Malan Hospital, Petrolina, Brazil; 3 Departments of Medicine, Obstetrics and Health Policy Management Evaluation, University of Toronto, St. Michael’s Hospital, 30 Bond Street, Toronto, ON M5B 1 W8, Canada

**Keywords:** Magnesium, Pregnancy, Prevention, Preeclampsia, Hypertension, Placenta, Perinatal, Preterm birth, Prematurity, Small for gestational age, Stillbirth

## Abstract

**Background:**

Preterm birth is the leading cause of infant mortality globally, including Brazil. We will evaluate whether oral magnesium citrate reduces the risk of placental dysfunction and its negative consequences for both the fetus and mother, which, in turn, should reduce the need for indicated preterm delivery.

**Methods/Design:**

We will complete a multicenter, randomized double-blind clinical trial comparing oral magnesium citrate 150 mg twice daily (n = 2000 women) to matched placebo (n = 1000 women), starting at 12^1/7^ to 20^6/7^ weeks gestation and continued until delivery. We will include women at higher risk for placental dysfunction, based on clinical factors from a prior pregnancy (e.g., prior preterm delivery, stillbirth or preeclampsia) or the current pregnancy (e.g., chronic hypertension, pre-pregnancy diabetes mellitus, maternal age > 35 years or pre-pregnancy maternal body mass index > 30 kg/m^2^). The primary perinatal outcome is a composite of preterm birth < 37 weeks gestation, stillbirth > 20 weeks gestation, neonatal death < 28 days, or SGA birthweight < 3rd percentile. The primary composite maternal outcome is preeclampsia arising < 37 weeks gestation, severe non-proteinuric hypertension arising < 37 weeks gestation, placental abruption, maternal stroke during pregnancy or ≤ 7 days after delivery, or maternal death during pregnancy or ≤ 7 days after delivery.

**Discussion:**

The results of this randomized clinical trial may be especially relevant in low and middle income countries that have high rates of prematurity and limited resources for acute newborn and maternal care.

**Trial registration:**

ClinicalTrials.gov Identifier NCT02032186, registered December 19, 2013.

## Background

### Prematurity as a serious public health problem

According to the World Health Organization (WHO), 15 million children each year are born preterm before 37 weeks gestation [1]. Prematurity is the leading cause of death in the neonatal period [2,3], and is the second leading cause of death in children aged 5 years and younger [1]. In low income countries, preterm birth before 32 weeks gestation has a mortality rate of 50% [1,2]. Among survivors, serious morbidity arises in the form of cerebral palsy and both visual and hearing impairment [4,5], which negatively impacts on the social and economic productivity of the family unit and the nation at large [6-8]. In the US, $26 billion are spent each year for the in-hospital care of the premature infant, a value that does not even include ongoing care and monitoring thereafter [6].

Globally, Brazil is among the top-10 countries with the largest number of premature births [1]. In 2011, among nearly three million births recorded in Brazil, 400,000 (13.6%) were preterm, contributing to almost half of the 40,000 infant deaths in that year [9].

### Prevention of preterm labor – the placenta as a target organ

The main forms of preterm delivery are either by *spontaneous mechanisms* (i.e., preterm labor with intact membranes or preterm premature rupture of membranes [PPROM]), or by way of intervention – *provider initiated* (“*medically indicated”*) *preterm delivery*” – in response to a maternal condition (e.g., preeclampsia), or a fetal indication (e.g., small for gestational age [SGA] fetal weight) [10]. Provider initiated preterm birth accounts for 41%, followed by spontaneous preterm labor (32%) and PPROM (27%) [11]. Among U.S. women whose first pregnancy resulted in a provider initiated preterm birth, there was a much higher subsequent chance of preterm birth by medical indication in the second pregnancy (odds ratio [OR] 10.6, 95% confidence interval [CI] 10.1-12.4), but less so if it followed a prior spontaneous preterm birth (OR 1.6, 95% CI 1.3-2.1) [12].

Two major factors appear to increase neonatal morbidity and mortality in the presence of preterm birth: i) SGA below the expected 10th percentile, and ii) the hypertensive disorders of pregnancy. Both factors especially potentiate the risk in relation to a provider initiated (medically indicated preterm birth).

In the EUROPOP Study, 23% of preterm infants born at 22 to 36 weeks gestation were SGA (adjusted OR, 2.33, 95% CI 2.09-2.60) [13]. Moreover, upon restricting their analysis to women with provider-initiated preterm birth, the effect of SGA was even greater (OR 6.38, 95% CI 5.47-7.45). The latter is especially noteworthy, since preterm interruption of pregnancies is a substantial and growing cause of preterm delivery in Brazil [14]. In the 2004 Pelotas birth cohort, 45% of Brazilian preterm birth were spontaneous and 55% were by indicated vaginal induction or Cesarean section [11]. Brazilian neonates delivered at late preterm, and who are also SGA, have higher morbidity than late preterm infants whose weight is appropriate for gestational age [15].

In a population-based study 97,000 live born singleton infants born to nulliparous Swedish women, there was a pronounced association between the hypertensive disease of pregnancy and SGA, especially for preeclampsia resulting in preterm delivery ≤ 32 weeks (OR of SGA: 40.5, 95% CI 31.5-51.4) and at 33–36 weeks (OR of SGA: 17.4, 95% CI 15.7-19.3) [16].

As noted by others, preterm delivery of the SGA fetus is especially pronounced in the co-presence of severe hypertensive disorders of pregnancy [17], of which 66% of preterm birth are provider initiated [18]. In a study from the Southern Brazil, for example, treated hypertension was associated with an OR of 2.74 (95% CI 1.78-4.22) for preterm birth, specifically among low-income mothers [10].

**Figure 1:**
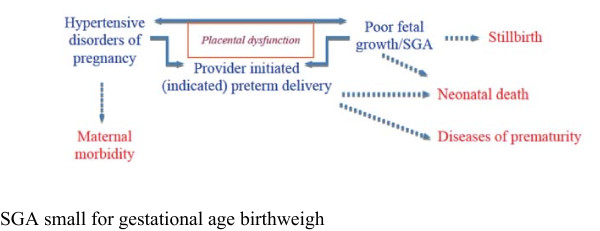
Placental dysfunction and its negative consequences for a woman mother and her fetus/newborn.

While both SGA and maternal hypertension may necessitate provider-initiated (“indicated”) preterm delivery [18-20] it is understood that placental dysfunction may be a major mediator of all three [21], as shown in Figure 1.

Placental dysfunction may result in adverse maternal clinical outcomes, namely, preeclampsia, placental abruption & placental infarction [22,23], as well as adverse perinatal outcomes, namely, stillbirth, poor fetal growth and preterm birth [24]. In Brazil, the national rate of stillbirths is 9.5 per 1000 births, while the corresponding rate is 27 per 1000 births in Northeast Brazil [25]. A significant percentage of stillbirths are related to nutritional deficiencies, and placental dysfunction is believed to be a cause of many, especially those stillbirths occurring preterm [26].

Placental dysfunction is particularly apparent when preeclampsia and preterm delivery occur concomitantly [27]. Interestingly, Mg^++^ has an immediate effect on placental vascular flow and reduced placental vascular flow is at least, in part, responsible for placental insufficiency and fetal intra-uterus growth restriction [28].

### Rationale for a preventive clinical trial of oral Mg^++^ citrate in pregnancy

We require a preventive strategy that attenuates and/or delays the development of placental dysfunction, and thus, decreases the onset of SGA and/or hypertensive disorders of pregnancy. In turn, this should reduce the need for preterm induction of labor or Cesarean delivery (i.e., provider initiated preterm delivery). Improving placental health should reduce the risk of antepartum and/or intrapartum stillbirth.

Although present in grains, green vegetables and seeds, insufficient Mg^++^ intake is common, especially in low-income regions. Adolescents and women are more prone to Mg^++^ deficiency [29]. It is recommended that women consume 280 mg of Mg^++^ per day [30], increasing in pregnancy [31]. Most Mg^++^ (99%) is intra-cellular, such that serum levels have a low accuracy for Mg^++^ deficiency [32]. Total and ionized Mg^++^ are inversely associated with gestational age in pregnancy [33]. Mg^++^ deficiency in pregnancy has been associated with a higher risk of chronic hypertension, preeclampsia, placental dysfunction and premature labor [34].

Oral Mg^++^ supplementation has been evaluated in pregnancy in a minor number of randomized controlled trials (RCT) (Table 1). A meta-analysis included seven RCTs, comprising 2,689 pregnant women [35]. In 6 RCTs, women were randomly allocated to an oral Mg^++^ supplement vs. a control group; the seventh study was a cluster RCT. Starting oral Mg^++^ supplementation before 25 weeks gestation was associated with a lower risk of PTB (RR 0.73, 95% CI 0.57-0.94). There was a lower risk of SGA (RR 0.70, 95% CI 0.53 to 0.93), fewer hospitalizations during pregnancy (RR 0.66, 95% CI 0.49 to 0.89) and fewer cases of antepartum hemorrhage (RR 0.38, 95% CI 0.16 to 0.90) [35]. While Mg^++^ supplementation reduced the risk of preeclampsia by 13%, this effect was not significantly so. However, since preterm onset of preeclampsia or severe preeclampsia was not specifically analyzed in the meta-analysis, few women had that outcome assessed, so little can be deduced about the impact of Mg^++^ supplementation on this relevant outcome. Moreover, it was not clear if the observed 27% relative risk reduction for preterm birth was predominantly a reduction in spontaneous vs. provider initiated preterm birth.

**Table 1:** Pooled data from randomized clinical trials of oral magnesium supplementation in pregnancy for the prevention of adverse pregnancy outcomes

**Outcome**	**Number of participants (RCTs) included**	**Control event rate (per 1000)**	**Magnesium supplementation event rate (per 1000)**	**Relative risk reduction,% (95% confidence interval)**
Preterm birth < 37 weeks gestation	2275 (5)	105	67	27 (6 to 43)
Preeclampsia	474 (2)	167	145	13 (−32 to 43)
Small for gestational age birthweight < 10 percentile	1741 (3)	119	83	30 (7 to 47)

Based on reference [35].

More recent data suggest that maternal Mg^++^ supplementation in pregnancy may have other perinatal benefits. In a double-blind RCT, 4,494 South African black pregnant women of low socioeconomic status were randomized to receive daily 128 mg Mg^++^ stearate slow-release or matching placebo [36]. Treatment was begun after 22 weeks gestation in most women, and was sustained for a mean of about 28 days in both groups. The rate of preterm delivery was 11.4% in the placebo group and 11.7% in the Mg^++^ group. The risk of the primary outcome of hypoxic-ischemic encephalopathy was non-significantly lower in the Mg^++^ group (OR 0.70, 95% CI 0.36 to 1.35), but the overall event rate was lower than expected in both groups. Interestingly, the risk of 3rd-trimester stillbirth was lower in Mg^++^ arm (OR 0.32, 95% CI 0.12 to 0.87). The late initiation and limited duration of Mg^++^ supplementation may have limited the evaluation of the efficacy of Mg^++^ in this RCT.

As stated by others, there is not enough high quality evidence to show that Mg^++^ supplementation during pregnancy is beneficial [35]. Certainly, what is needed is an RCT that enrolls enough women at risk, at an early enough gestational age, for the remaining duration of the pregnancy, and that can assess both perinatal and maternal outcomes that matter, including not only preterm birth, but also SGA and preterm preeclampsia. Our proposed RCT will address these goals.

### The proposed intervention: oral Mg^++^ citrate supplementation in pregnancy

Mg^++^ citrate is a widely used Mg^++^ supplement: it is inexpensive, easily absorbed and rarely has a detectable laxative effect. It can be taken as a powder, or placed into standard-dose capsules. Mg^++^ citrate has high solubility (55%) in water, in all states of gastric acid secretion. As demonstrated in one RCT, Mg^++^ citrate appears to be the most bioavailable of all oral Mg^++^ preparations [37]. Thus, oral Mg^++^ citrate is cheap, accessible, easy to manufacture. Thus, a programme of its use in pregnancy in low-middle income countries would be easy to initiate.

## Methods/Design

### Study objectives and design

#### Primary objectives

i. In women with 1 or more risk factors for an adverse pregnancy outcome, to determine whether there is a reduction in the **composite *perinatal *** **outcome** -- preterm birth before 37 weeks gestation, stillbirth after 20 weeks gestation, neonatal death before 28 days after birth, or SGA birthweight under the 3rd percentile -- following the administration of oral Mg^++^ citrate 150 mg twice daily versus oral placebo twice daily.

ii. In women with 1 or more risk factors for an adverse pregnancy outcome, to determine whether there is a reduction in the **composite *maternal *** **outcome** -- preeclampsia or eclampsia arising before 37 weeks gestation, severe non-proteinuric hypertension arising before 37 weeks gestation, placental abruption, maternal stroke during pregnancy or ≤ 7 days after delivery, or maternal death during pregnancy or ≤ 7 days after delivery -- following the administration of oral Mg^++^ citrate 150 mg twice daily versus oral placebo twice daily.

#### Secondary objectives

i. In women with 1 or more risk factors for an adverse pregnancy outcome, to determine whether there is a reduction in any single component of the **composite *perinatal *** **outcome** -- preterm birth before 37 weeks gestation, stillbirth after 20 weeks gestation, neonatal death before 28 days after birth, or SGA birthweight under the 3rd percentile -- following the administration of oral Mg^++^ citrate 150 mg twice daily versus oral placebo twice daily.

ii. In women with 1 or more risk factors for an adverse pregnancy outcome, to determine whether there is a reduction in any single component of the **composite *maternal *** **outcome** -- preeclampsia or eclampsia arising before 37 weeks gestation, severe non-proteinuric hypertension arising before 37 weeks gestation, placental abruption, maternal stroke during pregnancy or ≤ 7 days after delivery, or maternal death during pregnancy or ≤ 7 days after delivery -- following the administration of oral Mg^++^ citrate 150 mg twice daily versus oral placebo twice daily.

#### Study design

We will complete a multicenter double-blind, placebo-controlled randomized superiority clinical trial of oral Mg^++^ citrate supplementation.

#### Setting

The study will be done at two major centres: The first centre is the Instituto de Medicina Integral Prof. Fernando Figueira (IMIP), Recife-Pernambuco. IMIP maintains the largest hospital in Brazil exclusively dedicated to SUS. IMIP has 1,032 beds and performs more than 2,000 clinical daily visits. IMIP enrolls about 6,000 deliveries per year and has an obstetric intensive care unit. The second centre is the Dom Malan Hospital (HDM), Petrolina-Pernambuco. HDM assists the population of about 1 million people in 55 municipalities of Pernambuco, Bahia and Piauí States. HDM performs about 600 deliveries per month.

#### Participant inclusion and exclusion criteria

The following maternal inclusion criteria must all be met:

• Age 18–45 years at the time of enrollment,

• Gestational age at 12^1/7^ to 20^6/7^ weeks,

• Accurate estimated date of confinement, based on the last menstrual period among women with a regular menstrual cycle, or by a first-trimester pregnancy dating ultrasound,

• Singleton pregnancy,

• Current place of residence is within Recife or Petrolina,

• *One or more* of the following risk factors related to either:

A prior pregnancy

1. Preterm delivery at 24^1/7^ to < 36^6/7^ weeks

2. Stillbirth at > 20^1/7^ weeks gestation

3. Placental abruption

4. Preeclampsia or eclampsia

5. Liveborn infant with SGA < 10th percentile

6. Liveborn infant with birthweight < 2500 grams

The current pregnancy

1. Nulliparity

2. Chronic hypertension

3. Type 1 or type 2 diabetes mellitus

4. Maternal age > 35 years

5. Obesity (pre-pregnancy maternal body mass index > 30 kg/m^2^)

6. Currently smoking cigarettes

Any one of the following constitutes a reason for exclusion from the trial, determined at initial consideration for eligibility:

• Known uncontrolled hyperthyroidism

• Known active parathyroid disease of any kind

• Chronic kidney disease, defined by an estimate glomerular filtration rate under 60 mL/min/1.73 m^2^, as determined at baseline entry or by known history

• Chronic diarrheal disease

• High serum Mg^++^ concentration > 9.5 mmol/dL, as determined at baseline entry.

#### The intervention

We active arm is Mg^++^ citrate capsules (150 mg elemental Mg^++^ citrate per capsule), and the control arm is matched placebo capsules. Both will be manufactured by IMIP’s Department of Pharmacology, and will be identical in colour and shape. The 150 mg twice daily dose was chosen with the aim of achieving daily Mg^++^ needs without causing excess of Mg^++^ circulating levels. The study medication packages will be supplied to each local pharmacy with sequential numbers. Code break envelopes will be supplied to the lead pharmacist, but will be not available for the investigative team. Each pack will be individually prescribed for each participant.

Compliance/adherence, adverse events, and clinical intercurrences will be monitored by the research team at each routine prenatal visit until the completion of the treatment. Adherence will be defined as the ingestion of at least 80% of the prescribed dose.

#### Study outcomes

The individual components of the **composite *perinatal *** **outcome** -- preterm birth before 37 weeks gestation, stillbirth after 20 weeks gestation, neonatal death before 28 days after birth, or SGA birthweight under the 3rd percentile – are listed in Table 2. The individual components of the **composite *maternal *** **outcome** -- preeclampsia or eclampsia arising before 37 weeks gestation, severe non-proteinuric hypertension arising before 37 weeks gestation, placental abruption, maternal stroke during pregnancy or ≤ 7 days after delivery, or maternal death during pregnancy or ≤ 7 days after delivery – are listed in Table 2.

**Table 2:** Study outcomes, expected results and key indicators

**Study outcomes**	**Expected result**	**Key indicator**
***1a. Perinatal composite outcome (Main perinatal outcome)***	Reduction in the rate of the composite perinatal outcome among infants of women exposed to Mg^++^ vs. placebo	Preterm birth < 37 weeks gestation, stillbirth > 20 weeks gestation, neonatal death < 28 days after birth, or SGA birthweight < 10 percentile
1b. Preterm birth (PTB) (Secondary objective)	Reduction in the rate of PTB among infants of women exposed to Mg^++^ vs. placebo	Birth at gestational age < 37 weeks
1c. Stillbirth (Secondary objective)	Reduction in the rate of stillbirths among pregnant women exposed to Mg^++^	Fetal loss after 20 weeks gestation, in the absence of a major congenital anomaly evident at birth
1d. Small for gestational age birthweight < 10th percentile (Secondary objective)	Reduction in the rate of SGA among infants of women exposed to Mg^++^ vs. placebo	SGA detected by a birthweight < 10th percentile
1e. Neonatal death < 28 days after birth (Secondary objective)	Reduction in the rate of neonatal death SGA among infants of women exposed to Mg^++^ vs. placebo	Neonatal death of a liveborn infant from the date of birth up to and including 27 days after birth, in the absence of a major congenital anomaly evident at birth
1f. Neonatal intensive care unit (NICU) admission (Secondary objective)	Reduction in the rate of NICU admission among infants of women exposed to Mg^++^ vs. placebo	NICU admission < 28 days after birth
***2a. Maternal composite outcome (Main maternal outcome)***	Reduction in the rate of the composite maternal outcome among women exposed to Mg^++^ vs. placebo	Preeclampsia or eclampsia < 37 weeks gestation, severe gestational hypertension < 37 weeks gestation, placental abruption in pregnancy, or maternal stroke or death during pregnancy or ≤ 7 days after delivery
2b. Preeclampsia and eclampsia < 37 weeks gestation (Secondary objective)	Reduction in rate of preterm preeclampsia or eclampsia among pregnant women exposed to Mg^++^ vs. placebo	Increased blood pressure > 140/90 mm Hg associated with ≥ 2 proteinuria, and/or seizures, and/or the HELLP Syndrome, arising < 37 weeks gestation
2c. Severe non-proteinuric hypertension < 37 weeks gestation (Secondary objective)	Reduction in rate of preterm severe non-proteinuric hypertension among pregnant women exposed to Mg^++^ vs. placebo	Increased systolic blood pressure > 160 mm Hg or diastolic blood pressure > 105 mm Hg, with ≤ 1+ proteinuria, arising < 37 weeks gestation
2d. Maternal stroke (Secondary objective)	Reduction in rate of maternal stroke among pregnant women exposed to Mg^++^ vs. placebo	Abrupt onset of a focal neurological deficit in the distribution of a brain artery persisting more than 24 hours due to intracerebral hemorrhage or ischemic infarction, arising during pregnancy or ≤ 7 days after delivery
2d. Maternal intensive care unit (ICU) admission (Secondary objective)	Reduction in the rate of maternal ICU admission among women exposed to Mg^++^ vs. placebo	Adult ICU admission during pregnancy or ≤ 7 days after delivery

#### Randomization scheme

Participants will be randomized in a 2:1 fashion to either Mg^++^ citrate or placebo. Block randomization will be used to ensure a balanced number of participants in each group at any time during the study.

#### Data analysis and sample size

All outcomes will be analyzed by intention to treat (ITT), using logistic regression analysis. The main effects sizes for each outcome will be expressed as a rate (95% CI) and odds ratio (95% CI).

At the time of study entry, on the day of randomization, all women will undergo a measured serum Mg^++^ and creatine concentration. Any woman whose serum Mg^++^ concentration is over 9.5 mmol/dL, or whose estimated glomerular filtration rate (eGFR is under 60 mL/min/1.73 m^2^, will be excluded from the trial, and will not be counted in the ITT analysis. Otherwise, all other randomized participants will be included in the ITT analysis regardless of whether they complete the trial.

An efficacy analysis (on-treatment) analysis will be done for each study outcome, wherein women who were adherent with at least 80% of their dispensed tablets will be analyzed.

At a sample size of 1000 women assigned to placebo and 2000 women assigned to Mg^++^ citrate, with power of 80% and a 2-sided P-value of 0.05, we will be able to detect at least a 22% relative risk reduction in the primary perinatal composite outcome, assuming a primary perinatal composite outcome rate of 18% in the placebo group and 14% in the Mg^++^ group. In Recife there are 25,000 births a year. Of these 25,000, at least 30% (7,500) would attend one of the study prenatal clinics, & of those 7,500, 60% (4,500) would meet ≥1 of the eligibility criteria. With a participation rate of 50%, 2,250 women can be recruited in a year, thus requiring 1.25 years to recruit all women, and 2 years ascertain all primary outcome events.

#### Safety

The US FDA raised concern about prolonged Mg^++^ use in pregnancy (http://www.fda.gov/downloads/Drugs/DrugSafety/UCM353335.pdf). This was prompted by 18 reported cases of fetal and neonatal bone demineralization and fractures following long-term in utero exposure to intravenous over a mean of 9.6 weeks, at a mean total cumulative maternal dose of 3700 g. This adverse outcome was not seen in prior RCTs of 24–48 hour intravenous Mg^++^ sulphate for fetal neuroprotection (n = 6145 infants) [38,39] or for the prevention of eclampsia in mothers with preeclampsia (n = 11,444 women, including 3283 children followed to 18 months of age) [40]. The latter level I evidence does not negate the risk of prolonged intravenous Mg^++^ to fetal bone development, but the level IV data about potential harm are sparse and reflect very high doses of intravenous Mg^++^ sulphate.

At high intravenous doses of Mg^++^ sulphate, maternal side effects (nausea, cutaneous flushing, lassitude and muscle weakness) are common [38-40], essentially bordering between a therapeutic vs. a toxic effect. Mg^++^ intoxication causes a reduction in serum calcium concentration, leading to rapid decline in maternal serum parathyroid hormone (PTH) concentration – that is, hypocalcaemia may be partly due to the suppressive effects of acute hypermagnesaemia on PTH secretion [41]. Intravenous Mg^++^ sulphate crosses the placental barrier, which may neuroprotective effect the very preterm fetus, including a reduction in the risk of cerebral palsy, without increasing the risk of perinatal death [40,42]. While 300 mg of Mg^++^ citrate contains 12.4 mmol of Mg^++^, a fair amount is not absorbed, whereas intravenous MgS04 has near-100% bioavailability. With oral Mg^++^ therapy, the normal serum Mg^++^ level is 0.75 to 0.95 mmol/L. In one RCT of 4 weeks of supplemental oral Mg^++^oxalate (400 mg twice daily) in middle-aged non-pregnant adults, serum Mg^++^ levels only changed from 0.84 to 0.89 mmol/L [43]. In a second RCT of 16 weeks of Mg^++^ chloride (2500 mg per day) among non-pregnant adults with diabetes mellitus, serum Mg^++^ levels changed from 0.64 to 0.74 mmol/L [44]. In contrast, among pregnant women who receive intravenous Mg^++^ sulphate – whether as a tocolytic agent in preterm labor, for preeclampsia or for fetal neuroprotection – serum Mg^++^ levels rapidly rise to sustained concentrations of 2.0 mmol/L or more [45,46]. Certainly, acute neonatal depression is positively correlated with maternal serum Mg^++^ concentration in women administered intravenous Mg^++^ sulphate [47]. It is highly unlikely that our proposed dose of Mg^++^citrate 150 mg twice daily can increase maternal (or fetal) serum Mg^++^ concentration to even 50% of that seen when intravenous Mg^++^ sulphate is used in pregnancy.

One of the main determinants of maternal Mg^++^ handling is renal function, including after oral Mg^++^ administration [47,48]. Thus, as a safety measure in our proposed RCT, we will measure maternal serum creatinine concentration at 12–20 weeks gestation. We will deem ineligible any woman whose estimate glomerular filtration rate (eGFR) is under 60 mL/min/1.73 m^2^.

In terms of infant safety, we will assess for the presence of hypocalcemia at birth, defined as a total serum calcium concentration in the umbilical cord below 10 mg/dL. These measures will be obtained in all neonates of BRAMAG enrolled mothers who deliver within IMIP and the Dom Malan Hospital. We will also assess for the presence of neonatal osteopenia by plain X-ray examination of all newborns admitted to the neonatal intensive care unit (and who receive a routine chest X-ray as part of their care). It is expected that around 5% of all newborns studied (150/3,000) will have a chest X-ray. Radiological markers of osteopenia will be assessed on these plain radiographs by a radiologist.

## Discussion

We expect that oral Mg^++^ citrate supplements will lower the risk of preterm birth, perinatal mortality and neonatal morbidity, SGA, and will also positively impact on maternal morbidity and mortality. Thus, the intervention might not only save lives, but lower maternal and early childhood disability.

## Competing interests

The authors declare that they have no competing interests.

## Authors’ contributions

JGBA: study concept, drafting of manuscript, manuscript revision, approval of final version. JGR: study concept, drafting of manuscript, manuscript revision, approval of final version. CAFLA: study concept, approval of final version. IEAP: study concept, approval of final version. ACG: study concept, approval of final version.

## Pre-publication history

The pre-publication history for this paper can be accessed here:

http://www.biomedcentral.com/1471-2393/14/222/prepub

## References

[B1] Preterm birth http://www.who.int/mediacentre/factsheets/fs363/en/

[B2] Rubens CE Gravett MG Victora CG Nunes TM GAPPS Review Group Global report on preterm birth and stillbirth (7 of 7): mobilizing resources to accelerate innovative solutions (Global Action Agenda) BMC Pregnancy Childbirth 2010 10 Suppl 1 S7 20233388 10.1186/1471-2393-10-S1-S7 PMC2841775

[B3] Muglia LJ Katz M The enigma of spontaneous preterm birth N Engl J Med 2010 362 529 535 20147718 10.1056/NEJMra0904308

[B4] Simhan HN Caritis SN Prevention of preterm delivery N Engl J Med 2007 357 477 487 17671256 10.1056/NEJMra050435

[B5] Romero R Espinoza J Kusanovic JP Gotsch F Hassan S Erez O Chaiworapongsa T Mazor M The preterm parturition syndrome BJOG 2006 113 17 42 17206962 10.1111/j.1471-0528.2006.01120.x PMC7062298

[B6] Seubert DE Huang WM Wasserman-Hoff R Medical legal issues in the prevention of prematurity Clin Perinatol 2007 34 309 318 17572237 10.1016/j.clp.2007.03.008

[B7] Hubinont C Debieve F Prevention of preterm labour: 2011 update on tocolysis J Pregnancy 2011 2011 941057 22175022 10.1155/2011/941057 PMC3228310

[B8] Flood K Malone FD Prevention of preterm birth Semin Fetal Neonatal Med 2012 17 58 63 21893439 10.1016/j.siny.2011.08.001

[B9] Estatísticas vitais http://www2.datasus.gov.br/DATASUS/index.php?area=0205

[B10] Blencowe H Cousens S Oestergaard MZ Chou D Moller AB Narwal R Adler A Vera Garcia C Rohde S Say L Lawn JE National, regional, and worldwide estimates of preterm birth rates in the year 2010 with time trends since 1990 for selected countries: a systematic analysis and implications Lancet 2012 379 2162 2172 22682464 10.1016/S0140-6736(12)60820-4

[B11] Henderson JJ McWilliam OA Newnham JP Pennell CE Maternal factors associated with three phenotypes: spontaneous preterm labour, preterm pre-labour rupture of membranes and medically indicated preterm birth J Matern Fetal Neonatal Med 2012 25 642 647 21827362 10.3109/14767058.2011.597899

[B12] Ananth CV Getahun D Peltier MR Salihu HM Vintzileos AM Recurrence of spontaneous versus medically indicated preterm birth Am J Obstet Gynecol 2006 195 643 650 16949395 10.1016/j.ajog.2006.05.022

[B13] Zeitlin J Ancel PY Saurel-Cubizolles MJ Papiernik E The relationship between intrauterine growth restriction and preterm delivery: an empirical approach using data from a European case–control study BJOG 2000 107 750 758 10847231 10.1111/j.1471-0528.2000.tb13336.x

[B14] Barros FC Victora CG Matijasevich A Santos IS Horta BL Silveira MF Barros AJ Preterm births, low birth weight, and intrauterine growth restriction in three birth cohorts in Southern Brazil: 1982, 1993 and 2004 Cad Saude Publica 2008 24 Suppl 3 S390 S398 18797714 10.1590/s0102-311x2008001500004

[B15] Silveira MF Victora CG Barros AJ Santos IS Matijasevich A Barros FC Determinants of preterm birth: Pelotas, Rio Grande do Sul State, Brazil, 2004 birth cohort Cad Saude Publica 2010 26 185 194 20209222 10.1590/s0102-311x2010000100019

[B16] Clausson B Cnattingius S Axelsson O Preterm and term births of small for gestational age infants: a population-based study of risk factors among nulliparous women Br J Obstet Gynaecol 1998 105 1011 1017 9763054 10.1111/j.1471-0528.1998.tb10266.x

[B17] Hershkovitz R Erez O Sheiner E Bashiri A Furman B Shoham-Vardi I Mazor M Comparison study between induced and spontaneous term and preterm births of small-for-gestational-age neonates Eur J Obstet Gynecol Reprod Biol 2001 97 141 146 11451538 10.1016/s0301-2115(00)00517-0

[B18] Kase BA Carreno CA Blackell SC Sibai BM The impact of medically indicated and spontaneous preterm birth among hypertensive women Am J Perinatol 2013 30 843 848 23359232 10.1055/s-0033-1333676

[B19] McCowan LM Bulst RG North RA Gamble G Perinatal morbidity in chronic hypertension Br J Obstet Gynaecol 1996 103 123 129 8616127 10.1111/j.1471-0528.1996.tb09662.x

[B20] Palo P Erkkola R Risk factors and deliveries associated with preterm, severely small for gestational age fetuses Am J Perinatol 1993 10 88 91 8442812 10.1055/s-2007-994712

[B21] Groom KM North RA Poppe KK Sadler L McCowan LM The association between customised small for gestational age infants and pre-eclampsia or gestational hypertension varies with gestation at delivery BJOG 2007 114 478 484 17378821 10.1111/j.1471-0528.2007.01277.x

[B22] Ray JG Vermeulen MJ Schull MJ Redelmeier DA Cardiovascular health after maternal placental syndromes (CHAMPS): population-based retrospective cohort study Lancet 2005 366 1797 1803 16298217 10.1016/S0140-6736(05)67726-4

[B23] Roberts DJ Post MD The placenta in pre-eclampsia and intrauterine growth restriction J Clin Pathol 2008 61 1254 1260 18641412 10.1136/jcp.2008.055236

[B24] Salafia CM Vogel CA Vintzileos AM Bantham KF Pezzullo J Silberman L Placental pathologic findings in preterm birth Am J Obstet Gynecol 1991 165 934 938 1951558 10.1016/0002-9378(91)90443-u

[B25] Ferraz EM Gray RH A case–control study of stillbirths in northeast Brazil Int J Gynaecol Obstet 1991 34 13 19 1671015 10.1016/0020-7292(91)90532-a

[B26] Bring HS Varli IA Kublickas M Papadogiannakis N Petersson K Causes of stillbirth at different gestational ages in singleton pregnancies Acta Obstet Gynecol Scand 2014 93 86 92 24117104 10.1111/aogs.12278

[B27] Salafia CM Pezzullo JC López-Zeno JA Simmens S Minior VK Vintzileos AM Placental pathologic features of preterm preeclampsia Am J Obstet Gynecol 1995 173 1097 1105 7485300 10.1016/0002-9378(95)91333-5

[B28] Bernal AL The regulation of uterine relaxation Semin Cell Dev Biol 2007 18 340 347 17582797 10.1016/j.semcdb.2007.05.002

[B29] King DE Mainous AG Geesey ME Woolson RF Dietary magnesium and C-reactive protein levels J Am Coll Nutr 2005 24 166 171 15930481 10.1080/07315724.2005.10719461

[B30] Nordic Council of Ministers Nordic Nutrition Recommendations Scand J Nutr 1996 40 161 165

[B31] Standing Committee on the Scientific Evaluation of Dietary Reference Intakes, Food and Nutrition Board, Institute of Medicine Dietary Reference Intakes for Calcium, Phosphorus, Magnesium, Vitamin D, And Fluoride National Academies Pressin press 23115811

[B32] Mittendorf R Dambrosia J Dammann O Pryde PG Lee KS Ben-Ami TE Yousefzadeh D Association between maternal serum ionized magnesium levels at delivery and neonatal intraventricular hemorrhage J Pediatr 2002 140 540 546 12032519 10.1067/mpd.2002.123283

[B33] Arikan GM Panzitt T Gücer F Scholz HS Reinisch S Haas J Weiss PA Course of maternal serum magnesium levels in low-risk gestations and in preterm labor and delivery Fetal Diagn Ther 1999 14 332 336 10640870 10.1159/000020952

[B34] Wynn A Wynn M Magnesium and other nutrient deficiencies as possible causes of hypertension and low birthweight Nutr Health 1988 6 69 88 3072500 10.1177/026010608800600201

[B35] Makrides M Crowther CA Magnesium supplementation in pregnancy Cochrane Database Syst Rev 2001 4 CD000937 11687087 10.1002/14651858.CD000937

[B36] Harrison V Fawcus S Jordaan E Magnesium supplementation and perinatal hypoxia: outcome of a parallel group randomised trial in pregnancy BJOG 2007 114 994 1002 17578470 10.1111/j.1471-0528.2007.01409.x

[B37] Walker AF Marakis G Christie S Byng M Mg citrate found more bioavailable than other Mg preparations in a randomised, double-blind study Magnes Res 2003 16 183 191 14596323

[B38] Doyle LW Crowther CA Middleton P Marret S Rouse D Magnesium sulphate for women at risk of preterm birth for neuroprotection of the fetus Cochrane Database Syst Rev 2009 1 CD004661 19160238 10.1002/14651858.CD004661.pub3

[B39] Costantine MM Weiner SJ Eunice Kennedy Shriver National Institute of Child Health and Human Development Maternal-Fetal Medicine Units Network Effects of antenatal exposure to magnesium sulfate on neuroprotection and mortality in preterm infants: a meta-analysis Obstet Gynecol 2009 114 354 364 19622997 10.1097/AOG.0b013e3181ae98c2 PMC2761069

[B40] Duley L Gülmezoglu AM Henderson-Smart DJ Chou D Magnesium sulphate and other anticonvulsants for women with pre-eclampsia Cochrane Database Syst Rev 2010 11 CD000025 21069663 10.1002/14651858.CD000025.pub2 PMC7061250

[B41] Rocha VS Lavanda I Nakano EY Ruano R Zugaib M Colli C Calcium and magnesium status is not impaired in pregnant women Nutr Res 2012 32 542 546 22901563 10.1016/j.nutres.2012.05.010

[B42] Cholst IN Steinberg SF Tropper PJ Fox HE Segre GV Bilezikian JP The influence of hypermagnesemia on serum calcium and parathyroid hormone levels in human subjects N Engl J Med 1984 310 1221 1225 6709029 10.1056/NEJM198405103101904

[B43] Kawano Y Matsuoka H Takishita S Omae T Effects of magnesium supplementation in hypertensive patients: assessment by office, home, and ambulatory blood pressures Hypertension 1998 32 260 265 9719052 10.1161/01.hyp.32.2.260

[B44] Rodríguez-Morán M Guerrero-Romero F Oral magnesium supplementation improves insulin sensitivity and metabolic control in type 2 diabetic subjects: a randomized double-blind controlled trial Diabetes Care 2003 26 1147 1152 12663588 10.2337/diacare.26.4.1147

[B45] Lurie S Gur D Sadan O Glezerman M Relationship between uterine contractions and serum magnesium levels in patients treated for threatened preterm labour with intravenous magnesium sulphate J Obstet Gynecol 2004 24 247 248 15203617 10.1080/01443610410001660715

[B46] Lu JF Nightingale CH Magnesium sulfate in eclampsia and pre-eclampsia: pharmacokinetic principles Clin Pharmacokinet 2000 38 305 314 10803454 10.2165/00003088-200038040-00002

[B47] Robinson RR Murdaugh HV Jr Peschel E Renal factors responsible for the hypermagnesemia of renal disease J Lab Clin Med 1959 53 572 576 13654895

[B48] Simchen MJ Dulitzky M Mashiach S Friedman SA Schiff E Adjustment of magnesium sulfate infusion rate in patients with preterm labor Am J Obstet Gynecol 1998 179 994 998 9790387 10.1016/s0002-9378(98)70205-4

